# Determinants and Strategies for Implementation of the HOPE Self-Sampling Cervical Screening Intervention for Women Living with HIV in Ghana: A Context Assessment and Implementation Mapping Study

**DOI:** 10.21203/rs.3.rs-10695564/v1

**Published:** 2026-09-08

**Authors:** Nadia Adjoa Sam-Agudu, Karen Awura-Adjoa Ronke Coker, Emily Frueh, Obed Cudjoe, Sebastian Ken-Amoah, Nancy Innocentia Ebu Enyan, Gloria F. Nuer-Allornuvor, Emmanuel Abrefah, Alex Boadi Dankyi, Patrick Kafui Akakpo, Dorcas Obiri-Yeboah, Matthew Asare

**Affiliations:** University of Minnesota Medical School; University of Minnesota Medical School; Baylor University; University of Cape Coast; University of Cape Coast; University of Cape Coast; Cape Coast Teaching Hospital; Baylor University; University of Cape Coast; University of Cape Coast; University of Cape Coast; Baylor University

**Keywords:** Early detection of cancer, uterine cervical neoplasms, implementation support strategies, implementation science, HIV, Ghana

## Abstract

**Background:**

In Ghana, fewer than 5% of women eligible for cervical screening access this intervention. This screening gap has significant implications for early detection and treatment, especially for women living with HIV (WLWH), who are at higher risk of developing and dying from cervical cancer.

The HOPE-in-Ghana (HOPE-inG) study aimed to identify determinants of implementation for the HOPE self-sampling cervical screening intervention in Ghana’s Central Region and to select implementation strategies to optimize its implementation in this context.

**Methods:**

We applied the nominal group technique in a rapid context assessment with four purposively sampled stakeholder groups. Participants independently generated ideas on determinants of HOPE implementation, shared their ideas, provided clarifications, and voted to prioritize them. The Consolidated Framework for Implementation Research (CFIR) was used to categorize nominal group data as follows: determinants relevant to the Innovation (HOPE intervention); Inner setting (health facility sites of implementation); Outer setting (policy); Individuals (intervention recipients, deliverers, and decision-makers) and the Implementation Process. Prioritized determinants judged addressable within the study’s scope were then mapped to implementation strategies listed in the Expert Recommendations for Implementing Change (ERIC) taxonomy and specified using published reporting guidance.

**Results:**

We conducted four nominal groups among 65 participants, including WLWH, healthcare workers, clinic managers and government officials that identified determinants largely concentrated in the CFIR Inner Setting, Outer Setting and Implementation Process domains. Analysis yielded 29 prioritized and addressable determinants (14 facilitators and 15 barriers) which were mapped to 24 strategies, with focus on training and educating stakeholders, using evaluative and iterative strategies, stakeholder engagement, and providing interactive assistance, leading to the development of targeted job aids.

**Conclusions:**

Bridging the gap between evidence and practice requires consideration of both context and stakeholder interests. Our context assessment highlighted key changes in policy, health facility staff and environment, and implementation processes as critical to the successful integration of HPV self-sampling into Ghana’s HIV program and health system. Accordingly, we identified relevant implementation strategies to inform actionable job aids. We will evaluate whether these implementation support strategies improve adoption, cost, fidelity, penetration, and sustainability of the HOPE cervical screening intervention in our study setting.

**Trial registration:**

ClinicalTrials.gov NCT06800664. Registered on 29 January 2025.

## INTRODUCTION

Cervical cancer (CC) is the fourth most common cancer among women globally and the leading cause of cancer death among women in African countries. An estimated 660,000 new cases and 350,000 deaths occurred worldwide in 2022, and approximately 94% of these deaths occurred in low- and middle-income countries [1, 2]. Persistent infection with high-risk human papillomavirus (HPV) is the cause of virtually all cervical cancer cases, with high-risk genotypes HPV 16, 18, and 45 accounting for the largest share of invasive disease globally [3, 4]. CC screening is conducted using either a Papanicolaou (Pap) smear or HPV DNA testing. A Pap smear involves collecting cervical cells for cytological examination to detect precancerous and cancerous abnormalities, whereas HPV testing identifies the presence of high-risk HPV genotypes associated with cervical carcinogenesis [1, 5]. Women screening positive for high-risk HPV types are then triaged to receive visual inspection of the cervix, cytology, or colposcopic evaluation to identify cervical precancerous lesions and determine the need for treatment, such as thermal ablation, cryotherapy, loop electrosurgical excision procedure (LEEP), or referral for management of invasive disease.

Despite the existence of high impact interventions, women in low- and middle-income countries continue to experience relatively high CC mortality rates. Reasons include weak health systems, limited screening infrastructure and capacity, affordability barriers, and delayed access to screening, diagnosis, and treatment [6]. Women living with HIV (WLWH) have a sixfold higher chance of developing CC than women without HIV [7], and this is a critical public health concern in high HIV burden countries in Africa. The World Health Organization (WHO) recommends nucleic acid-based HPV testing for CC screening among WLWH starting at age 25 years, and every 3 to 5 years thereafter [5]. In Ghana, estimates indicate that the prevalence of high risk HPV infection is 28% [8], and less than 5% of women eligible for cervical screening access these services [9]. This has significant implications for early detection and lifesaving treatment, especially for WLWH, who are at higher risk for both incidence and mortality. It is therefore a priority to identify health interventions and strategies for delivering these interventions to increase uptake of cervical screening for early detection.

Home-based tests have been developed for women to enable them to collect their own samples and decentralize facility-based screening, which may be inconvenient and/or embarrassing. Our team developed the HOme-based self-sampling for cervical cancer Prevention Education (HOPE) combination intervention and documented its acceptability and feasibility for HPV self-sampling among WLWH in Ghana [10, 11]. The three-component HOPE intervention comprises an HPV self-sampling test, theory-based behavioral change communication messaging, and patient navigation. A follow-up cluster randomized controlled trial is evaluating the effectiveness and implementation of the HOPE intervention in Ghana (HOPE-inG)[12]. HOPE-inG extends the HOPE intervention by retaining its three core components while introducing a fourth component which advances the concept of implementation support strategies (ISS). We define ISS as implementation strategies most relevant to implementation support, which involves translating research into practice [12]. Unlike broader implementation strategies that apply to many different actors and in a multiplicity of settings [13], ISS apply to health system-level implementers and operates within health facility implementation settings to facilitate health system adoption and long-term sustainment.

HPV self-sampling is a well-established evidence-based intervention for increasing cervical screening uptake in both high and low- and middle-income countries [14-16]. Our team has demonstrated the effectiveness of HPV self-sampling in increasing cervical screening uptake among WLWH in Ghana [11]. There is however little evidence on approaches for successful implementation, that is, implementation strategies for optimizing the delivery, spread, scale up and sustainability of self-sampling in health systems, especially in low- and middle-income countries. The HOPE-inG study aims to close this implementation gap by applying implementation science methods to evaluate implementation of the HOPE intervention in real-world settings.

Our guiding implementation science framework for the HOPE-inG study is the Exploration, Preparation, Implementation and Sustainment (EPIS) framework [17]. This framework has been widely used to guide implementation science focused projects for cervical cancer and a variety of other health conditions and interventions across Africa [18-27]. We have already completed the Exploration Phase, where the research team and relevant stakeholders assessed Ghana’s health system needs for a sustainable cervical screening program with high patient uptake [10-12]. This led to the development and piloting of the HOPE intervention. The Preparation Phase involves the assessment of determinants of implementation for the HOPE intervention, identification of implementation strategies to address these determinants, and the development of an implementation plan based on these findings.

For the HOPE-inG study’s Preparation Phase, our overall aim was to develop a culturally appropriate, evidence-based implementation plan and provider training materials to support the effective delivery of the HOPE intervention within Ghana’s health system [12]. Our specific objectives were to: 1) conduct a context assessment to identify determinants (barriers and facilitators) of implementation for the HOPE intervention, and 2) map the identified determinants to feasible implement strategies that would enhance the success of HOPE implementation in our study setting in Ghana. The identified implementation strategies would then be incorporated into an implementation plan for the study’s Implementation Phase.

## METHODS

### Study Design

We leveraged the nominal group technique to conduct a rapid context assessment and implementation mapping process to identify determinants and relevant implementation strategies for the HOPE intervention. The nominal group technique involves four main steps: 1) independent generation of ideas or responses, 2) round robin sharing of ideas, 3) clarifications, and 4) discussion with voting to prioritize the ideas [28]. We used the Consolidated Framework for Implementation Research (CFIR) to guide data analysis for the nominal groups, which provided a systematic and multi-level health system lens to the analysis. CFIR has five domains, namely: the Innovation (the HOPE intervention), Inner Setting (health facility as site of HOPE implementation), Outer Setting (relevant policy), Individuals (intervention recipients, deliverers, and decision-makers), and Implementation Process [29]. The determinants to be identified were to inform the selection of implementation strategies to be evaluated with the HOPE intervention in a type 2 hybrid effectiveness-implementation trial.

### Study Setting and Sites

The HOPE-inG study is being implemented at four district-level hospitals in Ghana’s Central Region: Saltpond Municipal Hospital, Swedru Government Hospital, Our Lady of Grace Hospital in Breman-Asikuma, and St. Francis Xavier Hospital in Assin Fosu (Fig. 1) The site selection process is described elsewhere [12]. These facilities operate within Ghana’s tiered national health system, which is overseen by the Ministry of Health and implemented through the Ghana Health Service and Christian Health Association of Ghana (CHAG). The health system comprises teaching hospitals such as the Cape Coast Teaching Hospital, district hospitals, polyclinics, and community-based health planning and services compounds [30]. Publicly owned district hospitals such as the Saltpond and Swedru sites serve as first-referral centers, providing essential medical, surgical, maternal, and HIV services under national guidelines. The study sites in Breman-Asikuma and Assin Fosu are part of the Christian Health Association of Ghana (CHAG) nationwide network of faith-based, not-for-profit facilities that operate as public–private partnerships. These facilities deliver an estimated 30–40% of Ghana’s healthcare services despite owning a relatively small proportion of national health infrastructure [31]. Their strong community presence and integration into district health systems make them critical partners in expanding access to HIV and cervical cancer services.

HIV care in Ghana follows the national guidelines, which recommend universal testing, immediate initiation of antiretroviral therapy (ART) for all persons living with HIV, routine viral load monitoring, and differentiated service delivery models [32]. District hospitals and CHAG facilities provide HIV testing, ART initiation, follow-up, and laboratory monitoring as part of standard care. Cervical screening is guided by national and WHO recommendations, with HPV DNA testing, Pap cytology, and visual inspection with acetic acid recognized as appropriate modalities [5]. WLWH typically access cervical screening through reproductive health or outpatient departments, and none of the HOPE-inG study sites previously offered integrated HIV–cervical screening or treatment services. The HOPE-inG intervention therefore introduced a new model of integrated care where none had existed previously.

### Participant Recruitment and Data Collection

The nominal groups were conducted on January 16 and 17, 2025. We recruited purposively selected adult participants from four key stakeholder groups, namely, WLWH (intervention recipients), healthcare workers (intervention deliverers) and clinic managers (facility-level decision-makers) and government officials (health system-level decision-makers). WLWH were recruited during routine ART clinic visits. Healthcare workers were identified by their clinic managers and supervisors. Clinic managers and government officials included both national and Central Regional level policymakers from the Ghana Health Service, specifically the National AIDS Control Programme and the Non-Communicable Disease Programme. These policymaker participants were contacted through phone calls and emails.

Nominal groups were conducted in private rooms by experienced facilitators: MA, DOY, NIEE and NASA. Each group responded to the guiding question: “What are the barriers regarding the successful adoption and implementation of the HOPE intervention by clinic managers and healthcare workers?” Participants were presented with ranking sheets to document and rank their individual responses. Thereafter, participants shared their individually top-ranked ideas in round-robin fashion, which were documented by a designated rapporteur participant, or a facilitator if necessary.

Then, the collated top-ranked ideas put forth by each member of the group were projected or narrated for group voting and prioritization. The ranking sheets were then collected for each group after each participant had documented their individual responses and the group had come to a consensus on their priority determinants. For the WLWH group, participants who were illiterate or semi-literate were assisted by facilitators in documenting their suggestions and expressing their votes in the ranking step. Sessions lasted between 90 and 120 minutes and were conducted in Fante and/or English, with facilitators translating where needed.

### Data Analysis and Implementation Mapping

After already completing task 1 and 2a, we completed tasks 2b and 3 of the five main implementation mapping tasks as described by Fernandez et al [33] (Fig. 2): (1) conduct needs assessment and identify stakeholders; and (2a) outline implementation and effectiveness outcomes (completed in the Exploration Phase [11, 12]); (2b) identify determinants; (3) select implementation strategies; (4) produce implementation protocols and materials (in Preparation Phase); and (5) evaluate implementation outcomes (in upcoming Implementation and Sustainment Phases). The top five ranked determinants for each group were collated and sorted into barriers and facilitators, which were further mapped to the five CFIR domains. Next, the principal investigators (MA, DOY, NASA) reviewed the determinant data to establish consensus on what was addressable by the HOPE-inG study within its scope of work, budget, and project timeline. We then conducted implementation mapping of the determinants deemed feasible to implementation strategies listed in the Expert Recommendations for Implementing Change (ERIC) [13].

Determinants were classified as addressable if they could plausibly be modified by actions within the study’s scope of work, budget and timeline; determinants requiring national policy change, statutory reform or resources outside the trial were classified as not addressable and were reported but not carried forward. Selected implementation strategies were named, defined and operationalized following published recommendations for specifying and reporting implementation strategies [34], including describing the actor(s), the action(s), the action target(s), the implementation outcome(s) addressed, and the justification [34].

## RESULTS

Among a total of 65 participants, we conducted four nominal groups: one each among WLWH (N = 16); healthcare workers (N = 16); clinic managers (N = 16) and government officials (N = 17) (Table 1).

Thirty-six top-ranked determinants comprising 18 barriers and 18 facilitators spanning all five CFIR domains were documented from the four nominal groups (Table 2). This exceeded the 20 expected (five per group) because some groups could not reach consensus on their top five and retained several tied determinants. Primary and secondary CFIR domains for the top-ranked determinants were clustered in the Inner Setting (15 mentions), the Outer Setting (13 mentions), and the Implementation Process (12 mentions), while Individuals (six mentions) and the Innovation itself (one mention) were less prominent.

After independent review by the three study principal investigators, with disagreements resolved through discussion until consensus, 29 priority determinants comprising 14 facilitators and 15 barriers were identified for mapping onto implementation strategies listed in the ERIC taxonomy. These priority determinants were presented to the HOPE-inG Stakeholder Advisory Board for their review in May 2025, once initial analysis was completed. This nine-member Board was convened at the beginning of the Preparation Phase and comprised two WLWH who were leaders of a network of persons living with HIV, two frontline healthcare workers serving as facility coordinators, the Programme Manager and the Head of Clinical Care at the National AIDS Control Programme, a representative from the Non-Communicable Disease Programme, the Regional HIV Coordinator, and the Head of Clinical Care at the Regional Health Directorate. The Board reviewed the results of the context assessment, examined the newly available data on determinants and discussed and approved the findings as reflective of the Central Region health system context that was assessed.

These 29 determinants are indicated in Table 2 as those addressable by the study. Mapping of the 29 priority determinants was conducted by five researchers (EF, OC, ABD, KAARC and NASA) and triangulated to yield 73 non-unique determinant-strategy pairings (24 addressing facilitators, 49 addressing barriers) representing a smaller number of discrete strategies. These implementation strategies were then grouped into the nine broad categories described by Waltz et al [35] (Fig. 3).

Thereafter, these implementation strategies were sorted to focus only on those that were relevant to implementers, i.e., healthcare workers and clinic managers who would be delivering the HOPE intervention in the Inner Setting at the study sites. The 24 implementation strategies meeting these criteria were the HOPE-inG study ISS. In our study, implementation support strategies also address or leverage EPIS bridging factors, which are connections between the Inner organizational and Outer funding/policy contexts. The majority of the 24 ISS identified were in the “Train and Educate Stakeholders” category (n = 7), followed by “Use Evaluative and Iterative Strategies” (n = 4) and “Develop Stakeholder Interrelationships” (n = 4) (Fig. 3).

For Task 4 of the implementation mapping process (produce implementation protocols and materials), we focused on developing job aids to support the translation of the 24 priority ISS into actionable and measurable tasks to improve implementation by clinic managers and healthcare workers. Our implementation outcomes of interest are derived from Proctor’s Implementation Outcomes Framework [36] and are the measures by which the impact of these ISS will be assessed (Table 3).

Table 4 presents all HOPE-inG ISS identified, along with their ERIC descriptions, implementation strategy categories, relevant job aids and implementation outcomes of interest. Of the 24 ISS identified, two implementation strategies were new and were not listed in the ERIC taxonomy: “Build workers’ skills to facilitate their motivation and retention”; and “Pay professional and lay staff consistently.” These were categorized under Waltz et al’s “Support Clinicians” and “Utilize Financial Strategies” categories, respectively [35].

The job aids are expected to guide implementation action to address multilevel determinants of HOPE implementation towards more successful effectiveness and implementation outcomes. These tools also address bridging factors, which comprise structures, processes and relationships that link the Inner and Outer Settings and help to move evidence into sustained practice [17](Table 5). The focus on bridging factors allows for health system bottlenecks at the external policy/community/health system and internal practice interfaces to be better recognized and addressed for successful integration and implementation of HIV and cervical cancer services.

## DISCUSSION

Our EPIS Preparation-phase assessment identified barriers and facilitators to HOPE implementation across all five CFIR domains. The concentration of determinants in the Inner Setting, Outer Setting and Implementation Process domains indicates that the organizational context, external policy and operational planning and execution are critical to the successful integration of HIV and cervical screening in our study setting [37, 38]. The identification of 73 candidate determinant-strategy pairings indicates considerable opportunity to address barriers and leverage facilitators. However, prioritizing addressable determinants and mapping them to feasible implementation strategies reflects a pragmatic approach to sustainable implementation planning and action [39].

Across stakeholder groups, several identified determinants reflected broader operational and health system constraints rather than concerns specific to the HOPE intervention itself. Key barriers included inadequate documentation and data management systems, shortages of supplies and logistics, low staff motivation, difficulties with post-self-sampling follow-up, and persistent misinformation. In contrast, stakeholders highlighted training, formal facility-level introduction of the intervention, integration with existing cancer programs, availability of suitable spaces for self-sampling, and standardized implementation tools as important facilitators. Collectively, these findings suggest that successful implementation of HOPE is likely to depend substantially on the capacity of health facilities and the broader health system to integrate the intervention into routine practice, support healthcare workers, and ensure effective coordination of screening, documentation, referral, and follow-up processes.

Rapid approaches and theory-driven implementation science methods provided efficiency and rigor to the context assessment and identification of implementation strategies in this study. Nominal groups enabled both rapid and prioritized identification of determinants among key stakeholders, an advantage over somewhat slower traditional qualitative research methods for assessing determinants. The CFIR framework supported our assessment of multi-level determinants to better understand their complex interplays across Inner and Outer settings in Ghana’s health system. EPIS’ key function in our study is to guide the process of planning and implementation of the HOPE intervention and bringing attention to bridging factor mechanisms that link the Inner Setting to the Outer Setting. This allowed us to make considerations for these potential implementation bottlenecks through relevant implementation support strategies to be evaluated in the Implementation and Sustainment Phases [17]. Implementation mapping provided a rigorous, theory-driven process for the selection of implementation strategies most relevant to the identified priority determinants.

Integration of HPV self-sampling into HIV care in our study setting is contextually appropriate but operationally complex [40]. The HOPE combination intervention (HPV self-sampling, the 3R communication model, and patient navigation) aims to address low cervical screening coverage among women in Ghana and the heightened risk faced by WLWH [10, 11]. Integrating screening into routine HIV care is expected to increase uptake and continuity of care, but requires alignment of clinic workflows, staff roles, supply chains, training, and referral/treatment pathways; these operational needs are likely to explain the multiplicity of implementation strategies identified [13, 40]. We considered these operational needs and made the decision to further develop job aids for HOPE-implementing healthcare workers and their supervising clinic managers. Job aids make complex tasks simpler, reduce cognitive load, and promote more consistent implementation with higher fidelity in different health systems across Africa [41-44]. The HOPE-inG job aids span a range of activities relevant to prioritized implementation strategies, from stakeholder engagement to training, facilitation, salary support, and sustainment. Evaluation of the implementation strategies whose actions are supported by the job aids will be conducted in the Implementation and Sustainment Phases; implementation outcomes of interest include adoption, cost, fidelity, penetration and sustainability.

During the Implementation phase, the 24 ISS and their corresponding job aids will guide context-responsive HOPE implementation. Training and support strategies are intended to support healthcare worker adoption and fidelity, while monitoring, feedback, stakeholder engagement, and implementation planning strategies will support integration of HOPE into routine service delivery. Evaluation of these strategies via the implementation outcomes of interest will help to assess whether the Preparation-phase decisions translate into implementation success during the trial.

Other studies in similar African settings have leveraged comparable implementation science approaches for CC prevention. Itanyi et al applied a virtual nominal group technique, implementation mapping and the EPIS framework to guide their context assessment for the ACCESS CC screen-and-treat study among WLWH in Nigeria [26, 45]. That process yielded 18 determinants, the most significant being human resource gaps, and poor access to, and lack of demand for CC services. The CHESS study is applying CFIR and the RE-AIM framework in its evaluation of mentor mother peer support in the integration of HPV self-sampling with HIV care for WLWH in Nigeria [46]. The Game Changers for Cervical Cancer Prevention study is applying EPIS and RE-AIM in the evaluation of a peer-led, group advocacy training intervention for increasing cervical screening uptake among women in Uganda [22]. The CarciSCAN study is a prospective observational cohort study that is also evaluating self-sampling for cervical screening in Ghana, in Bono East Region; however, it focuses on the general population and does not appear to be applying implementation science methods [47]. Together, these studies demonstrate the growing application of implementation science methods to CC prevention in African settings. HOPE-inG adds to this growing body of implementation science evidence on cervical cancer interventions and strategies in African countries.

### Implications for Implementation Planning and Research

Implementation science capacity and its applications are growing among African researchers, and more of these studies are being led by Africans in African settings. The rapid consensus-building and theory-based methods and practical feasibility considerations for the selected strategies allow for timely and rigorous development of our implementation plan and tools. The predominance of determinants at the organizational, policy, and implementation process levels underscores the importance of addressing health system determinants for successful implementation in our setting. The systematic approach described here could serve as a practical model for implementation planning, not only for cervical cancer prevention across Ghana, but also for other integrated health service interventions in similarly resource-constrained settings.

### Strengths and Limitations

Strengths of our study include the use of the nominal group technique for rapid consensus-building in the context assessment, application of theory-driven frameworks such as CFIR and EPIS, the use of an implementation mapping approach to identify implementation strategies, and the further effort to develop job aids to make the implementation strategies more actionable. Furthermore, the study’s focus on implementation support strategies aligns with the finding that determinants were concentrated in the Inner setting, in addition to the Outer Setting and Implementation Process. While the study employed rigorous methods and approaches, it has a few limitations. First, facilitator assistance with documentation and voting among WLWH with limited literacy, while necessary for equitable participation, may have introduced some influence on responses for those individual participants. However, the subsequent group vote and Stakeholder Advisory Board review and ratification are expected to add further validation to the group’s collective response. Second, the nominal groups had challenges in limiting their priority determinants, as such, the research team was presented with a longer list of determinants than expected. However, the team was able to address this issue through a review by the three principal investigators to focus only on determinants addressable within the study’s scope. Another limitation is that this is a single study in just one of Ghana’s 16 regions, as such, our findings may not be generalizable to all of Ghana and to other countries within or beyond Africa.

## CONCLUSIONS

Bridging the gap between evidence and practice requires that implementation context and stakeholder interests are taken into consideration. Findings from our context assessment indicated that successful integration of self-sampling cervical screening into Ghana’s health system would require key changes in policy, the health facility staff and environment, and implementation processes. Accordingly, we identified relevant implementation strategies and developed actionable job aids to promote consistent, high-fidelity implementation. The HOPE-inG study’s Preparation phase produced a pragmatic, stakeholder-informed set of prioritized determinants and implementation strategies to support successful implementation of the HOPE intervention in Ghana. Attention to facility-level adaptation, clear linkage-to-treatment pathways, and iterative monitoring will be essential to translate increased screening into reduced cervical cancer morbidity and mortality among WLWH. We have now embarked upon the Implementation Phase, where we are evaluating and will generate evidence on impact of these implementation support strategies on adoption, cost, fidelity, penetration and sustainability of the HOPE intervention in our study setting.

## Figures and Tables

**Figure 1:**
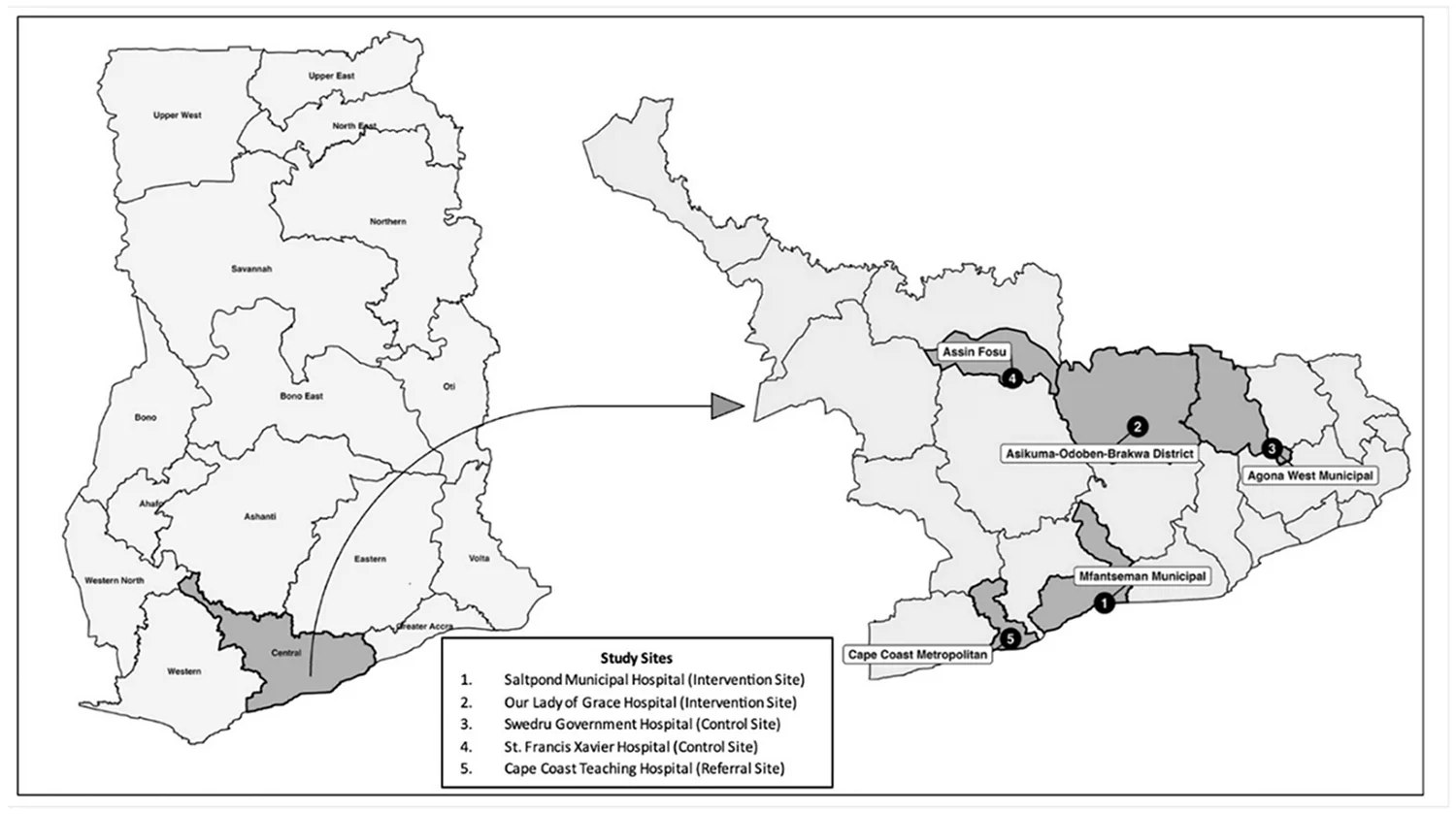
Map of Ghana with Central Region and HOPE-inG Study Sites

**Figure 2:**
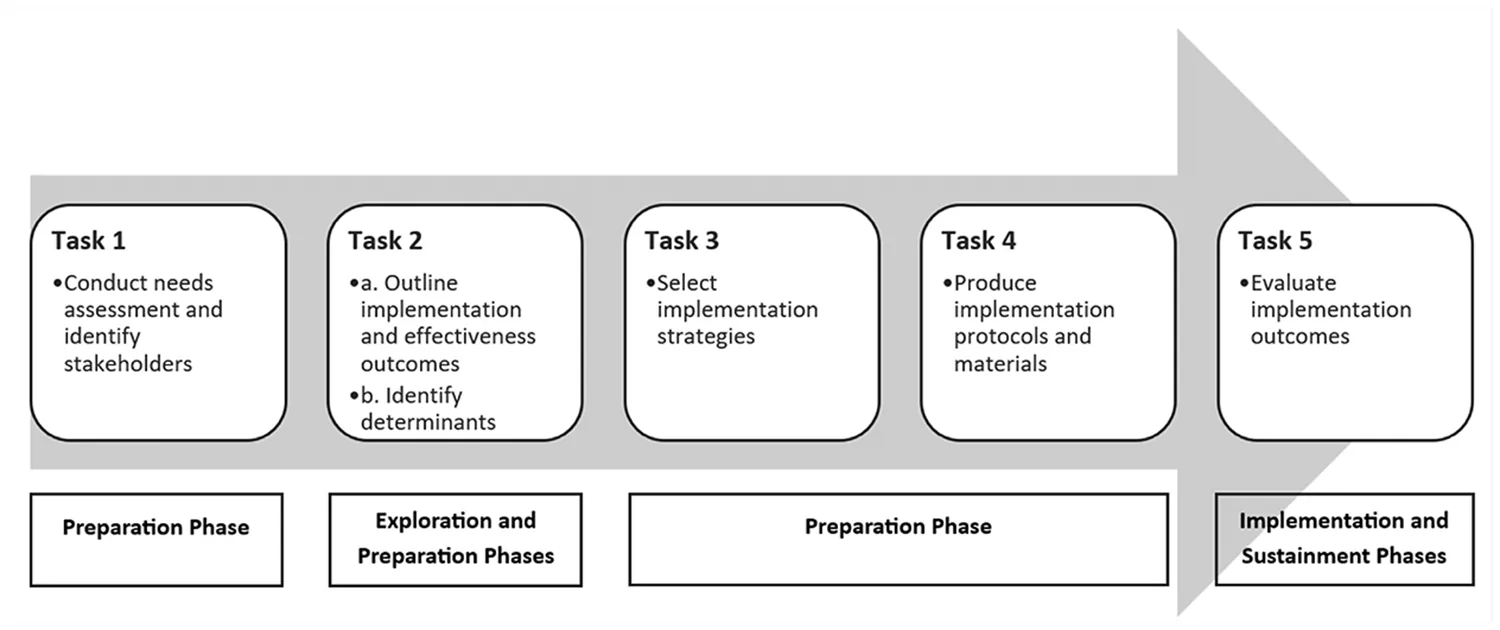
HOPE-inG Implementation Mapping Tasks through the four EPIS Phases

**Figure 3:**
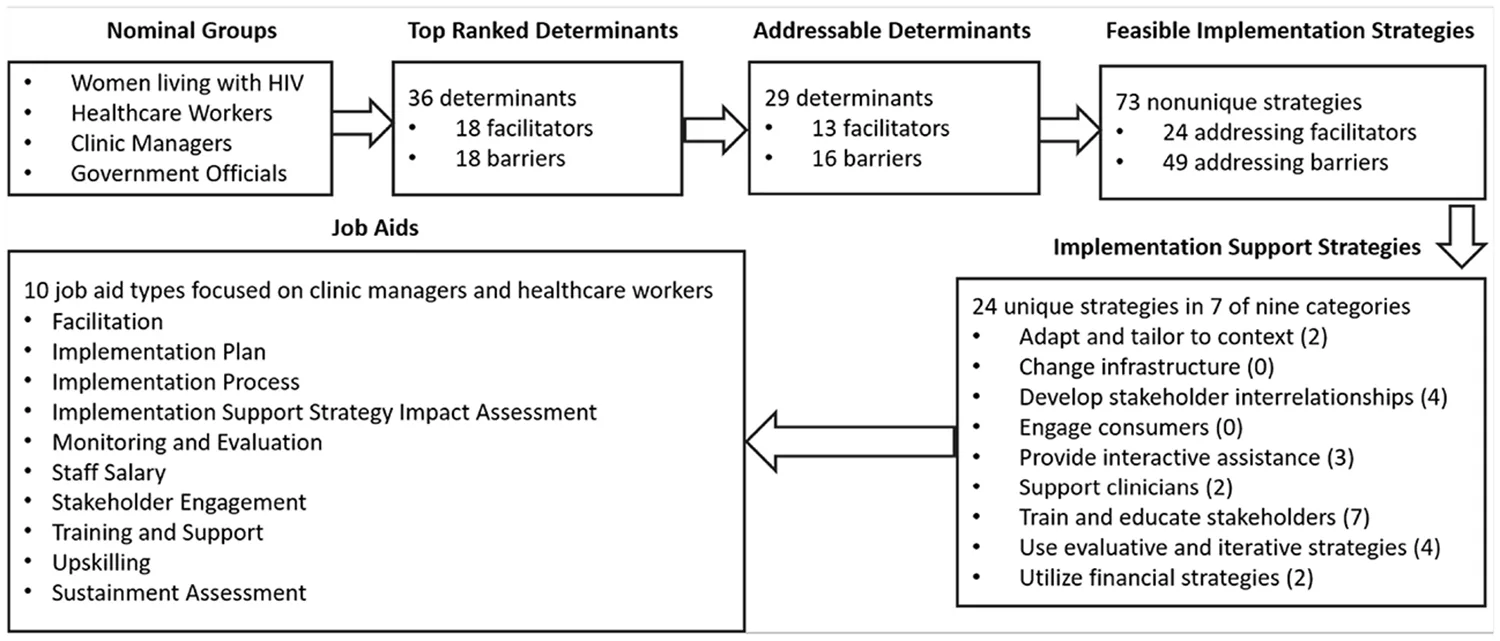
Identifying Determinants and Mapping Implementation Strategies in the Preparation Phase

**Table 1:** Nominal Group Participant Profiles

Nominal Group	Participants	N	% female	Age range	Tenure in current role
**Group 1**	**Women living with HIV**	16	100	21 to 55 yrs	Not applicable
**Group 2**	**Healthcare workers**	16	75	25 to > 54 yrs	< 1 year: 12.5%
	**Doctors**	*3*	*0*		1–5 yrs: 25.0%
	**Nurses/midwives**	*12*	*100*		6–10 yrs: 43.8%
	**Medical lab scientists**	*1*	*0*		> 10 yrs: 18.8%
**Group 3**	**Clinic managers**	16	63	25 to > 54 yrs	< 1 year: 0%
					1–5 yrs: 62.5%
					6–10 yrs: 12.5%
					> 10 years: 25.0%
**Group 4**	**Government officials**	17	53	35 to > 54 yrs	< 1 year: 11.8%
					1–5 yrs: 11.8%
					6–10 yrs: 47.1
					> 10 yrs: 29.4%

**Table 2:** Top Ranked Determinants from Nominal Groups Mapped onto CFIR Domains

Participant Group	CFIR Domain *(Primary listed* *before secondary* *domain if more than* *one domain applies)*	Determinant	Determinant Type	Addressable by Study?
**Women Living with HIV**	Outer Setting Implementation Process	Cervical screening should be added to antenatal screening tests.	Facilitator	Yes
	Inner Setting Individuals	Poor communication and unprofessional behavior from healthcare providers.	Barrier	Yes
	Implementation Process	Creating awareness about cervical cancer, its consequences, and the free screening and treatment should be emphasized during clinics and through peer-led initiatives.	Facilitator	Yes
	Outer Setting Inner Setting	Financial challenges coupled with abuse by implementers, such as surcharging clients, further worsen the difficulties clients face in accessing tests.	Barrier	Yes
	Inner Setting	Inadequate logistics	Barrier	Yes
	Inner Setting	Logistics for testing should be made available at facilities.	Facilitator	Yes
	Inner Setting	Lack of designated physical spaces for sample collection.	Barrier	Yes
	Inner Setting Individuals	Recruit nurses who are patient with clients and support them with more educational opportunities.	Facilitator	Yes
	Outer Setting Individuals	(Self)-stigmatization - familiar faces at the facility for clients.	Barrier	No
	Outer Setting	The government should provide subsidies, cover treatment costs through health insurance, and facilitate HPV vaccination processes.	Facilitator	No
**Healthcare Workers**	Outer Setting Implementation Process	Limited awareness creation leads to low community engagement and participation.	Barrier	Yes
	Outer Setting	Certification and Continuous Professional Development programs for healthcare workers.	Facilitator	No
	Outer Setting	Limited transportation options and long distances to facilities.	Barrier	Yes
	Inner Setting	Challenges in documentation and data management.	Barrier	Yes
	Implementation Process	Ineffective follow-up screening for home-screened participants.	Barrier	Yes
	Implementation Process	Launch the cervical screening program at the facilities.	Facilitator	Yes
	Outer Setting Implementation Process	Leverage on the pre-existing breast cancer program for cervical screening promotion.	Facilitator	Yes
	Inner Setting Individuals	Low staff motivation to sustain home visits.	Barrier	Yes
	Implementation Process	Proper information given (training) to the clinicians to be able to pass on to clients.	Facilitator	Yes
**Clinic Managers**	Inner Setting	Lack of efficient documentation and data management.	Barrier	Yes
	Implementation Process	Formally launch the new intervention at facilities.	Facilitator	Yes
	Outer Setting	Leverage on pre-existing programs like breast cancer during promotion.	Facilitator	Yes
	Individuals	Low staff motivation to sustain the project during home visits.	Barrier	Yes
	Inner Setting	Frequent shortages of logistics and supplies.	Barrier	Yes
	Implementation Process	Uncertainty around long-term project sustainability.	Barrier	Yes
	Implementation Process	Providing comprehensive training for healthcare workers, managers, and coordinators.	Facilitator	Yes
**Government Officials**	Inner Setting Individuals	Lack of effectiveness in trained staff	Barrier	No
	Inner Setting	Inadequate documentation and data capture by healthcare workers.	Barrier	Yes
	Outer Setting	Implementing a structured incentivization system for different cadres of healthcare workers and management.	Facilitator	No
	Inner Setting Outer Setting	Allowing management to recruit healthcare workers and ensuring clinical managers are supported by the National AIDS Control Program.	Facilitator	No
	Inner Setting	Clinic management should ensure that bathrooms where self-sampling will occur are clean and have the necessary supplies.	Facilitator	Yes
	Inner Setting	Self-sampling should be done at the facility, avoids contamination and preserves viability with respect to distance and time. Washrooms where samples will be taken should be carefully selected to avoid stigma.	Facilitator	Yes
	Implementation Process	Onsite training of healthcare workers	Facilitator	Yes
	Implementation Process	Provide standard operating procedure documents and job aids to clients and patient navigators.	Facilitator	Yes
	Outer Setting Innovation	Stigma associated with women living with HIV using self-sampling kits discourages participation and may necessitate alternative sampling methods.	Barrier	No
	Outer Setting	Misinformation is an issue especially regarding HIV, and any messaging should be addressed to the general population.	Barrier	Yes

**Table 3:** HOPE-inG Implementation Outcomes and Definitions

**1**	**Implementation Outcome**	**Study Definition [** 36 **]**
	**Adoption**	**The intention, initial decision, or action by healthcare workers and clinic management to implement the HOPE intervention.**
2	Cost	The cost impact of implementation of the HOPE intervention and its associated implementation strategies.
3	Fidelity	The degree to which the HOPE intervention is implemented as planned in the study protocol
4	Penetration	The level of integration of the HOPE intervention and established cervical screening practices within the study sites’ routine operations
5	Sustainability	The extent to which the HOPE intervention is independently maintained or institutionalized within the study sites’ routine operations by implementing staff.

**Table 4:** HOPE-inG Implementation Support Strategies, their Relevant Job Aids, and Implementation Outcomes of Interest

Implementation Support Strategy (ISS, N = 24) *(Bold: strategies for* *topmost ranked* *determinants)* (*non-ERIC strategy)		ERIC Description of the ISS (*description by *study team)*	Waltz et al Category *(7 of 9* *categories* *represented)*	Relevant Job Aids (N = 10)	Implementation Outcome(s) *(Listed in order of* *importance if* *more than one* *domain applies)*
1	Alter incentive, allowance structures	Work to incentivize the adoption and implementation of the clinical innovation	Utilize Financial Strategies	Staff Salary	Adoption Sustainability Cost
2	**Audit and provide feedback**	Collect and summarize clinical performance data over a specified time period and give it to healthcare workers and clinic managers to monitor, evaluate, and modify provider behavior	Use Evaluative/Iterative Strategies	Monitoring and Evaluation	Fidelity
3	Build a coalition	Recruit and cultivate relationships with partners in the implementation effort	Develop Stakeholder Relations	Stakeholder Engagement	Sustainability Penetration/Reach
4	**Build workers' skills to facilitate their motivation and retention**	Building workers’ implementation skills drives motivation and retention by satisfying intrinsic needs for autonomy, mastery, and purpose.*	Support Clinicians/Employees	Upskilling	Adoption Fidelity
5	Conduct cyclical small tests of change	Implement changes in a cyclical fashion using small tests of change before taking changes system-wide.	Train and Educate; Use Evaluative/Iterative Strategies	ISS Impact Assessment; Roles and Responsibilities; Sustainment Assessment	Fidelity Sustainability
6	**Conduct educational meetings**	Hold meetings targeted toward different stakeholder groups to teach them about the clinical innovation	Train and Educate	Implementation Process	Penetration/Reach Adoption
7	**Conduct ongoing training**	Plan for and conduct training in the clinical innovation in an ongoing way	Train and Educate	Training and Support	Fidelity Adoption
8	Develop a formal implementation blueprint	Develop a formal implementation blueprint that includes all goals and strategies. Use and update this plan to guide the implementation effort over time	Use Evaluative/Iterative Strategies; Develop Stakeholder Relations	Implementation Plan	Fidelity
9	Develop and implement tools for quality monitoring	Develop, test, and introduce into quality-monitoring systems the right input—that are often specific to the innovation being implemented	Use Evaluative/Iterative Strategies	Monitoring and Evaluation	Fidelity
10	Develop educational materials	Develop and format manuals, toolkits, and other supporting materials in ways that make it easier for stakeholders to learn about the innovation and for clinicians to learn how to deliver the clinical innovation	Train and Educate	Training and Support; Upskilling	Fidelity
11	Distribute educational materials	Distribute educational materials (including guidelines, manuals, and toolkits) in person, by mail, and/or electronically	Train and Educate	Training and Support; Upskilling	Fidelity
12	**Facilitation**	A process of interactive problem solving and support that occurs in a context of a recognized need for improvement and a supportive interpersonal relationship	Support Clinicians/Employees; Provide Interactive Assistance	Facilitation	Fidelity
13	**Identify and prepare champions**	Identify and prepare individuals who dedicate themselves to supporting, marketing, and driving through an implementation, overcoming indifference or resistance that the intervention may provoke in an organization	Support Clinicians/Employees; Develop Stakeholder Relations	Implementation Process	Fidelity Sustainability
14	Identify early adopters	Identify early adopters at the local site to learn from their experiences with the practice innovation	Develop Stakeholder Relations	Implementation Process	Adoption
15	Make training dynamic	Vary the information delivery methods to cater to different learning styles and work contexts, and shape the training in the innovation to be interactive	Support Clinicians/Employees	Training and Support; Upskilling	Fidelity
16	Pay professional/lay staff consistently*	Ensure consistent and timely payments of due salaries to HCWs and lay staff delivering cervical cancer services to sustain continuous service delivery and avoid strikes.	Utilize Financial Strategies	Staff Salary	Adoption Cost
17	Provide clinical supervision	Provide clinicians with ongoing supervision focusing on the innovation. Provide training for clinical supervisors who will supervise clinicians who provide the innovation	Support Clinicians/Employees; Provide Interactive Assistance	Training and Support; Upskilling; Monitoring and Evaluation	Fidelity
18	Provide local technical assistance	Develop and use a system to deliver technical assistance focused on implementation issues using local personnel	Provide Interactive Assistance	Facilitation; Implementation Process	Fidelity
19	Provide ongoing consultation	Provide ongoing consultation with one or more experts in the strategies used to support implementing the innovation	Support Clinicians/Employees	Training and Support	Adoption Fidelity
20	Remind clinicians	Develop reminder systems designed to help clinicians to recall information and/or prompt them to use the clinical innovation	Support Clinicians/Employees	Implementation Process	Adoption Fidelity
21	Use advisory boards and workgroups	Create and engage a formal group of multi-disciplinary stakeholders to provide input and advice on implementation efforts and to elicit recommendations for improvements	Train and Educate	Stakeholder Engagement	Sustainability
22	**Use data experts**	Involve, hire, and/or consult experts to inform management on the use of data generated by implementation efforts	Adapt and Tailor to Context	Monitoring and Evaluation	Fidelity
23	**Use data warehousing techniques**	Integrate clinical records across facilities and organizations to facilitate implementation across systems	Adapt and Tailor to Context	Monitoring and Evaluation	Fidelity
24	Use train-the-trainer strategies	Train designated clinicians or organizations to train others in the clinical innovation	Train and Educate; Support Clinicians/Employees	Training and Support	Adoption Fidelity

**Table 5:** Addressing Bridging Factors for Cervical Cancer Prevention with HOPE-inG Job Aids

Inner Organizational Setting	Bridging Factor Category	Relevant HOPE- inG Job Aids	How Job Aids Bridge Inner-Outer Setting	Outer Policy and External/Community Setting
	Policy–Practice Linkages	• Implementation Process • Implementation Plan • Sustainment Assessment	• Translate national HIV and cancer prevention guidelines into facility workflows • Ensure long-term alignment with policy goals	
	Partnerships, Stakeholder and Community Engagement	• Facilitation • Implementation Process • Stakeholder Engagement	• Link external experts with internal implementers to support implementation • Link patient communities to health services • Strengthen collaboration between public and faith-based facilities	
	Data Systems and Monitoring	• Monitoring and Evaluation	• Link facility data to national data monitoring • Ensure accuracy and accountability in translating policy to clinic practice • Provide feedback loops between national policy, provider efforts and patient outcomes data for continuous improvement	
	Technical Assistance and Capacity Building	• Training and Support • Upskilling • ISS Impact Assessment	• Build staff capacity to implement national policy through training • Link external experts with internal implementers to support implementation • Assess readiness and impact for scale-up	
	Staff Motivation and Incentives	• Staff Salary	• Align external funding streams with internal staff retention • Motivate providers to implement national policy and sustain integrated HIV and cervical cancer services

## Abbreviations

ART: antiretroviral therapy
CC: cervical cancer
CFIR: Consolidated Framework for Implementation Research
CHAG: Christian Health Association of Ghana
DNA: deoxyribonucleic acid
EPIS: Exploration, Preparation, Implementation and Sustainment
ERIC: Expert Recommendations for Implementing Change
HCW: healthcare worker
HIV: human immunodeficiency virus
HOPE: HOme-based self-sampling for cervical cancer Prevention Education
HOPE-inG: HOme-based self-sampling for cervical cancer Prevention Education in Ghana
HPV: human papillomavirus
ISS: implementation support strategies
LEEP: loop electrosurgical excision procedure
NCI: National Cancer Institute
NIH: National Institutes of Health
RE-AIM: Reach, Effectiveness, Adoption, Implementation, and Maintenance
WHO: World Health Organization
WLWH: women living with HIV
